# Study on the Characteristics of the Dispersion and Conductivity of Surfactants for the Nanofluids

**DOI:** 10.3390/nano12091537

**Published:** 2022-05-02

**Authors:** Sedong Kim

**Affiliations:** German Engineering Research and Development Center LSTME Busan Branch, Busan 46742, Korea; sedong.kim@lstme.org

**Keywords:** nanofluid, surfactant, heat transfer performance, dispersion, cellulose

## Abstract

Given the importance of nanofluid dispersion and stability, a number of approaches were proposed and applied to the nanofluid preparation process. Among these approaches, the noncovalent chemical process was intensively utilized because of its effective dispersion ability. For the noncovalent dispersion method, polymers and surfactants are typically used. In order to find an effective noncovalent dispersion method, several types of solutions were prepared in this study. The widely used naturally cellulose nanocrystal (CNC) aqueous solution was compared with several surfactant aqueous solutions. The dispersion characteristics of the prepared fluids were examined by UV/VIS spectroscopy at operating wavelengths ranging from 190 to 500 nm. Furthermore, the heat capacity and the electrical and thermal conductivity of the fluids were analyzed to evaluate their heat transfer performance and conductivity. The Lambda system was utilized for thermal conductivity measurement with operation at proper temperature ranges. The electrical conductivity of the fluids was measured by a conductivity meter. This experimental study revealed that the cellulose nanocrystal was an effective source of the noncovalent dispersion agent for thermal characteristics and was more eco-friendly than other surfactants. Moreover, cellulose aqueous solution can be used as a highly thermal efficient base fluid for nanofluid preparation.

## 1. Introduction

Nanofluid has been studied over the past decades and has a great potential to improve heat transfer properties [1]. However, unless the nanoparticles of the nanofluid are well dispersed, enhanced thermal performance [2,3] cannot be expected [4]. For this reason, two methods are generally applied for the well dispersion of nanofluids [5]; one is a mechanical method, and the other is a chemical method. However, the ultrasonic excitation and grinding of the mechanical method can leave too many fragments in a nanofluid when CNTs are used as nanoparticles. Therefore, it is not good to reduce the aspect ratio for the degree of dispersion stability [6]. This mechanical method is time-consuming and inefficient.

Unlike the mechanical method, covalent and non-covalent chemical methods [7,8,9,10] can avoid the aggregation of nanoparticles. The covalent methods have been functionalization with many chemical moieties to enhance the solubility of solvents; however, strong chemical synthesis at high temperatures causes defects on the carbon nanotube (CNT) surface, changing the electrical characteristics of CNT. On the other hand, the noncovalent method involves the adsorption of the chemical moieties onto the CNT surface, either through π-π stacking interactions, such as in DNA; uncharged surfactants; or the Coulomb attraction in the case of charged chemical moieties. The noncovalent method is effective in the sense that it does not alter the π-electron cloud of graphene, in turn protecting the electrical characteristics of nanotubes. For instance, polymers and surfactants are widely used for CNT dispersion through non-covalent methods.

Among those for CNT dispersion, cellulose is the richest polymer in nature. Over the past few years, the development of cellulose-based materials for CNT dispersion has been reported [11,12]. Cellulose nanocrystals (CNCs) have become widespread in many studies [13,14]. They are rod-shaped nanoparticles obtained from the acid hydrolysis of cellulose. They are about 10 to 100 nm in diameter and 100 to 1000 nm in length, depending on the cellulose source and the hydrolysis conditions.

CNC strongly interacts with the water molecules through hydrogen bonding because of the hydroxyl groups on the cellulose molecules [15,16]. Surfactants are classified into cationic, anionic, nonionic, and amphoteric depending on the charge of the head group [17,18,19,20]. In ionic surfactants, the surfaces of the particles have the same charge, which can generate electrostatic repulsion. Strong electrostatic repulsion between nanoparticles promotes the stable dispersion of nanofluid [21].

Many kinds of surfactants are used with nanoparticle suspensions, such as sodium dodecyl sulfate (SDS), sodium dodecyl benzenesulfonate (SDBS), and lauryl betaine (LB), to increase the dispersion and stability of nanofluids [22,23]. In order to choose the proper surfactant for a particular application, it is very important to conduct a systematic study of different parameters, such as stability and concentration [24,25,26,27,28]. Yurekli et al. [29] and Hertel et al. [30] studied changes in the phase behavior of CNTs on the basis of the concentration and the type of interaction of the surfactants. However, there have been fewer systematic studies on the different, proper parameters of cellulose [31,32,33] influencing the nanoparticle dispersion of a base fluid. 

In this study, the noncovalent process was conducted to maintain conductivity and enhance the nanoparticle dispersion for a base fluid, and [34,35] the heat capacity was also compared with that of other surfactants according to previous studies. It was found that cellulose is not only hydrophilic and eco-friendly, but it is also more thermally efficient than other surfactants. From these results, it can be confirmed that this study will make a significant contribution to the heat transfer technologies related to nanofluids because it shows good dispersion and stability to avoid nanoparticle agglomeration and identifies the stable thermal and electrical characteristics of a base fluid.

## 2. Materials and Methods

### 2.1. Materials

Demineralized water (DW) was prepared using a membrane-type DW device, which can produce DW with water quality of under 10 ppm of total dissolved solids (TDS). The cellulose nanocrystal (CNC) used in this research was extracted from the western hemlock plant and was supplied by SKB Tech, South Korea. Sodium dodecyl sulfate (SDS, CH_3_(CH_2_)11OSO_3_Na) with a 288.38 relative molecular mass (Junsei Chemical Co., Ltd, Tokyo, Japan); sodium dodecyl benzene sulfonate (SDBS, C_18_H_29_NaO_3_S), hard type, with a 348.48 relative molecular mass (Chemical Industry Co., Ltd, Tokyo, Japan), and dodecyl betaine (DB, C_16_H_33_NO_2_) with a 271.44 relative molecular mass (Avention Co., Ltd., Seoul, Korea) were used for the dispersants.

### 2.2. Methods

The electrical conductivity meter (Model CM-25R) used a contacting-type conductivity sensor, which consists of an electrode. The titanium–palladium alloy electrode was specifically sized and spaced to provide a known “cell constant”. A UV/Visible Spectrophotometer (X-ma 3000 Series Spectrophotometer, Human Co., Ltd., Seoul, Korea) was used to measure the dispersibility of the aqueous solution. The LAMBDA system measured the thermal conductivity of the aqueous fluids according to ASTM D 2717 by hot wire methods (evaluation of thermal conductivity of liquids). The heat capacities of all samples were measured by applying a constant heat source at the same time, and a comparison of the temperature of each surfactant was made. For all the measurements, tests were recorded at the same time, and each was carried out three times in the same way. In the repeated measurements, no significant differences were found.

### 2.3. Preparation of Samples

Garg et al. [36] experimentally studied the sedimentation and dispersion properties of nanofluid dispersed in a multi-walled carbon nanotube (MWCNT) over time. They reported that nanofluid treated with ultrasonic treatment for 40 min had a maximum thermal conductivity improvement effect.

**Figure 1:**
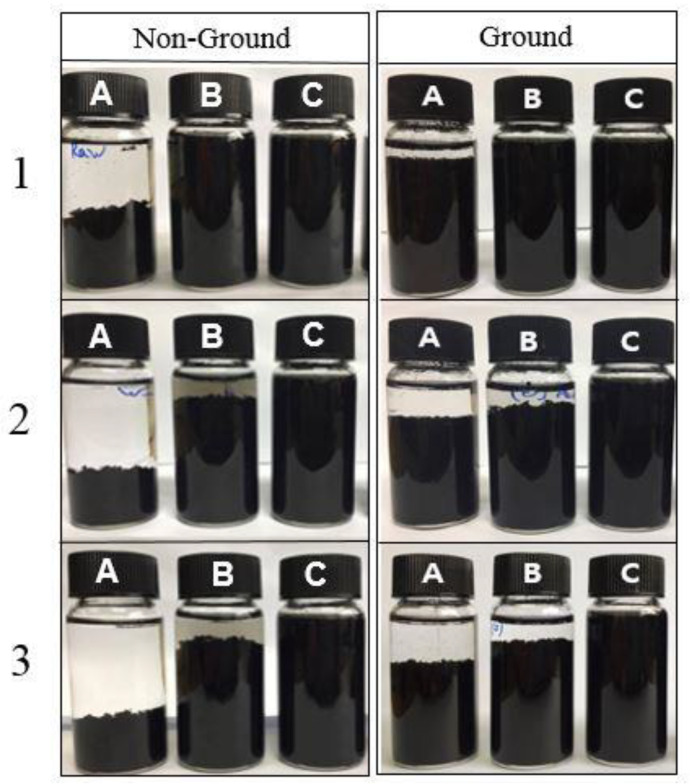
Sedimentation of the CNT nanofluid over time. (1) After sonication, (2) after 7 days, (3) after 30 days. Reprinted from ref. [37].

Figure 1 shows the sedimentation of the MWCNT nanofluid [37]. Following Garg et al., the cellulose and surfactants were dispersed with DW, and the excitation frequency and period were 42 kHz and 40 min, respectively. CNC was heated to temperatures ranging from 50 to 90 °C because CNC solution is not easily dispersed well without heating according to Molnes et al. [38]. All samples were prepared at a concentration of 0.1 wt%.

## 3. Results and Discussion

### 3.1. Structures of CNC

**Figure 2:**
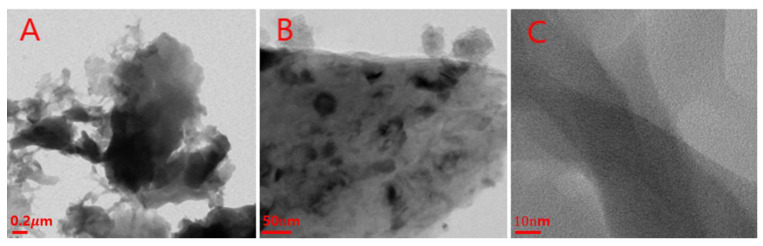
TEM image of cellulose nanocrystal ((**A**): 0.2 μm, (**B**): 50 nm, (**C**): 10 nm).

As seen in Figure 2, the morphological analysis of the cellulose was performed by Transmission Electron Microscopy (TEM, Technai 128 FEI). Figure 2A shows the agglomerations of nanocellulose formed as bundles by the strong hydrogen bonds between the single cellulose crystallites. However, Figure 2B,C show that the agglomerated particles consist of multiple single particles, which gathered together and formed the large aggregates. Through the TEM investigation, it can be seen that nanocellulose was formed by the aggregation of small rods.

### 3.2. Electrical Conductivity of Solution

In the area of electromechanical microelectronics, the property of electrical conductivity is an important factor. Some devices need electrical conductivity, while other devices do not need conductivity because of electrical interference. For instance, it is required for the probe to have a high thermal conductivity but low electrical conductivity.

Therefore, this study investigated the electrical conductivity of several factors. The calibration was performed with a potassium chloride standard (1.41, 12.86 μS/cm) solution before the measurement.

**Figure 3:**
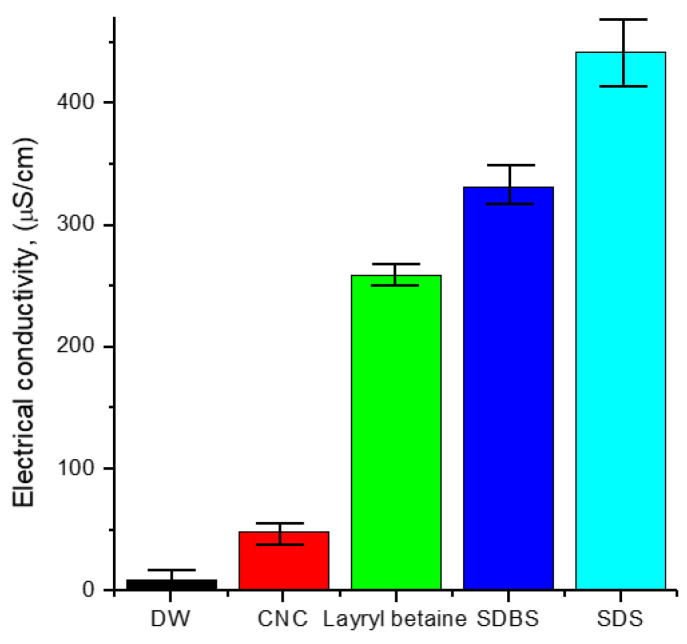
Electrical conductivity of each surfactant.

**Figure 4:**
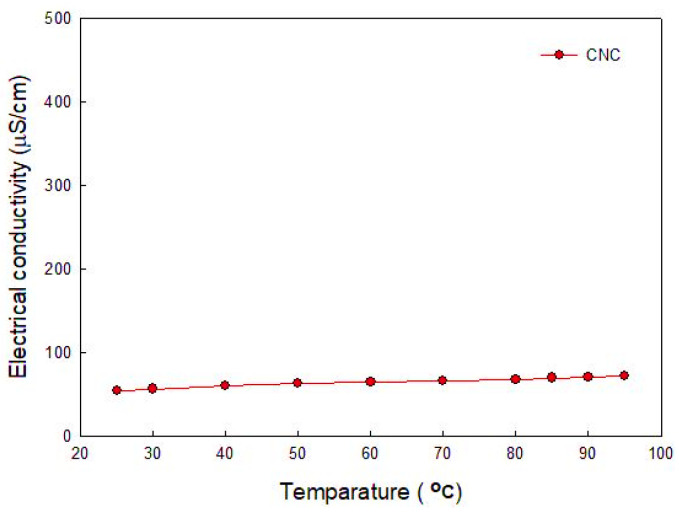
Electrical conductivity of CNC with increase in temperature.

As seen in Figure 3, the electrical conductivity of the CNC solution was lower than that of other surfactants. In the case of CNC, its electrical conductivity was not severely changed with the temperature variation, as shown in Figure 4, which means that CNC has the characteristic of low electrical conductivity regardless of the temperature variation.

In general, as the temperature increases, the electrical conductivity also increases in an aqueous solution because of fast ion diffusion. Therefore, most insulators can be combined with CNC regardless of temperature. It can be also applied in industrial fields in which the electrical conductivity of a working fluid can potentially be a threat to a system and its surroundings.

### 3.3. Dispersibility of Solution

In order to investigate the light absorbance characteristic of a solution in accordance with wavelength, a UV/VIS spectrophotometer was used. The UV/VIS spectrophotometer can measure the light absorbance of wavelengths ranging from 190 to 500 nm, which shows the light absorbance characteristics of the solutions.

The UV/VIS spectrophotometer is generally used for two purposes in nanofluid studies, which are the investigations of the concentration and the dispersion of nanoparticles. As the concentration of nanoparticles increases, the light absorbance increases. Additionally, the better the dispersion, the higher the light absorbance if the concentration of nanoparticles is the same.

**Figure 5:**
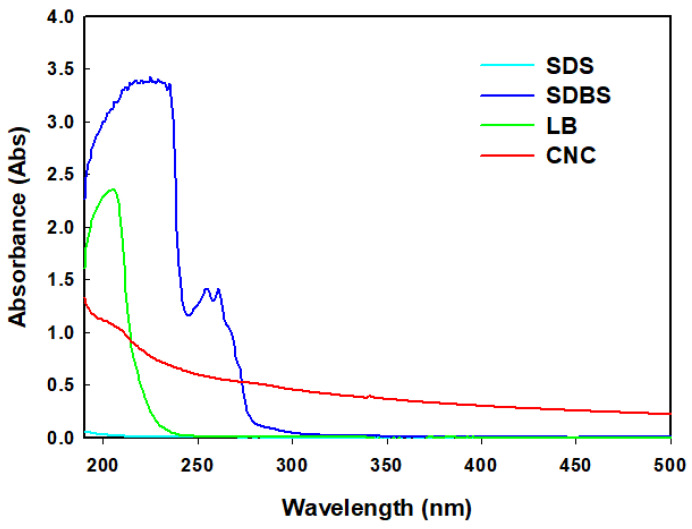
UV/VIS spectra of each surfactant.

Figure 5 presents the experimental results showing the light absorbance characteristics through the UV/VIS when each material was independently mixed with DW. Since each of the three surfactants and CNC were mixed with DW alone, Figure 4 provides the information on how the three surfactants affected the light absorbance when they were used for a well dispersion of nanoparticles.

Figure 5 shows that SDS does not absorb the lights over the measurement range of UV/VIS, which means that SDS is transparent in a solution and that SDS does not affect the light absorbance of a nanofluid. In the cases of SDBS and LB, some peaks were observed from 200 to 280 nm; however, those peaks fell into the range of ultraviolet rays. Therefore, they are also transparent when mixed with DW. Therefore, if three surfactants are used for a better dispersion of nanoparticles, the investigation of the visible light absorbance can be the index of a well dispersion, which means that the well dispersion can be roughly evaluated with the naked eye.

When CNC was independently mixed with DW, its light absorbance gradually decreased as the wavelength increased. Especially, it should be noticed that CNC has very low absorbance characteristics in the ultraviolet range compared with other surfactants, such as SDBS and LB. These results have an important physical meaning when making nanofluids.

**Figure 6:**
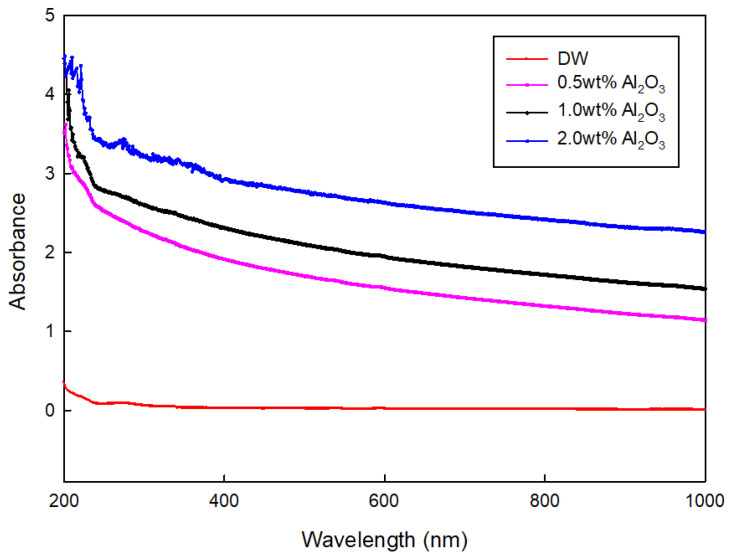
Absorbance of a base liquid (DW) and the nanofluids with three different alumina concentrations. Reprinted from ref. [5].

The CNC has smooth absorbance characteristics over the whole UV/VIS wavelength, which indicated that CNC could provide more detailed information on the dispersion and concentration. If SDBS and LB are used as the surfactants for the dispersion, it is difficult to use light absorbance in the ultraviolet range for the evaluation criteria of dispersion and concentration. Figure 6 shows the UV/VIS measurement for the Al_2_O_3_ nanofluid, which was previously performed by the author [5]. As seen in Figure 6, the smooth absorbance clearly provides the concentration variation, which means that CNC is a good surfactant to evaluate the dispersion and concentration of nanoparticles.

### 3.4. Thermal Conductivity of Solution

The thermal conductivities were measured by the LAMBDA measuring system to investigate each solution.

The LAMBDA system is based on the hot-wire method, and a platinum wire with a 0.1 mm diameter was applied as the hot wire. The detailed principles of this can be found in the previous research [39].

**Figure 7:**
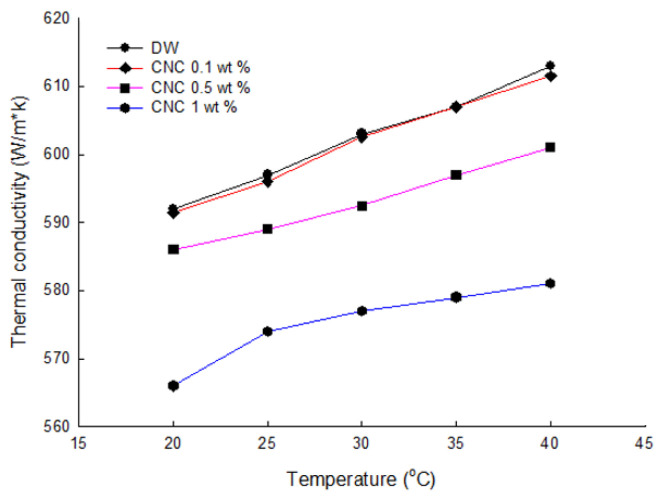
Thermal conductivity measurements of CNC according to concentration.

Figure 7 shows the thermal conductivities according to the concentration of CNC and the temperature variation. It can be seen that the thermal conductivity of CNC nanofluid decreased when the content of cellulose increased. It was reasonable to compare each surfactant at a concentration of 0.1% because when the CNC concentration was 0.1%, the value of thermal conductivity was high. Therefore, all samples were set at the proper concentration of nanofluid of 0.1 wt%.

**Figure 8:**
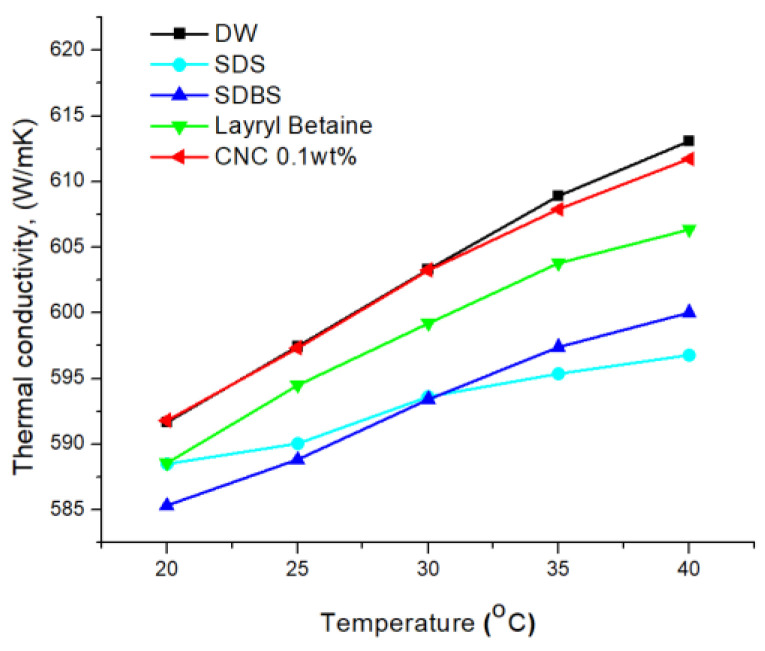
Thermal conductivity measurements of each surfactant.

As seen in Figure 8, CNC showed the highest thermal conductivity, followed by LB, and SDS and SDBS had similar values of thermal conductivity. Generally, when a surfactant is used in the preparation of a nanofluid, the thermal conductivity is degraded [15], and thus it may be seen that a good thermal conductivity value of cellulose is excellent for the preparation of a nanofluid.

### 3.5. Heat Capacity of Solution

Each sample was prepared for the same amount of solution and was constantly heated by a hot plate and magnetic stirrer, and the liquid temperatures were measured by thermally insulated T-type thermocouples. Each sample was measured for 20 min at the same initial temperature and same magnetic RPM. Temperatures were recorded in the data logger.

**Figure 9:**
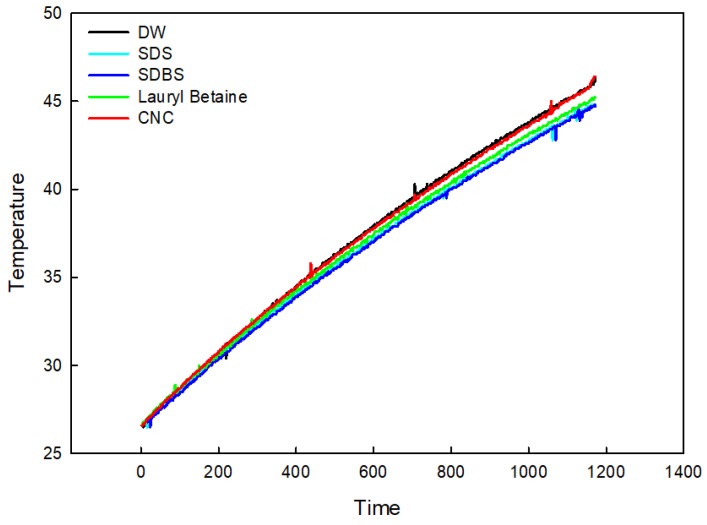
The comparison of heat capacity of various surfactants.

Figure 9 shows the result of the temperature variation of samples during the heating process. It is known that the heat capacity *C* of a sample is represented by the formula:

(1) $$C=\frac{Q}{\Delta T}$$

where *Q* is the heat supplied to the samples, and ΔT is the temperature variation during the heating process. It is possible to grasp the difference in heat capacity from the temperature variation in the samples. The measured heat capacities were the orders of SDS, SDBS, DB, and CNC, as seen in Figure 9. The fact that cellulose has a higher temperature increase rate than other surfactants shows that the heat transfer rate is more excellent than that of the others. It is concluded that CNC can be a good option as a dispersant for nanofluid because of its superior properties to other surfactants.

## 4. Conclusions

The importance of dispersion and stability in the application of nanofluid in industries is becoming more prominent. Accordingly, this study investigated an approach to the nanofluid manufacturing process.

Among these methods, effective dispersion ability was shown by intensively utilizing non-covalent methods rather than strong chemical methods. In this paper, cellulose and surfactants were generally used as non-covalent dispersion methods.

(1) The structure analysis of nano callouses was conducted by the TEM method. It showed rod-shaped nanoparticles acquired from the acid hydrolysis of callouses. The size of the CNC was about 100 nm in diameter and 1000 nm in length. It is shown in Figure 1 that CNC can interact with water strongly by hydrogen bonding because of the hydroxyl group on the molecule.
(2) The electrical conductivity of solutions was studied to figure out the electrical interference in the application of base fluid in industry. CNC had the lowest value of electrical conductivity compared with other surfactants. Furthermore, it was found that, unlike other surfactants, the electrical conductivity of CNC did not change with temperature.
(3) The absorbance of samples was investigated using a UV/VIS spectrophotometer. When other surfactants were used for dispersion, it was hard to use the light absorbance in the ultraviolet range for the evaluation criteria of dispersion and concentration. However, it was revealed that CNC has a stable value across different wavelengths, which indicates that CNC could provide more detailed information on the dispersion and concentration.
(4) The thermal conductivities were examined by the LAMBDA system. First, it was found that the low concentration had high thermal conductivity by comparing the thermal conductivity according to the CNC concentration. As the cellulose content increased, the thermal conductivity of the CNC nanofluid decreased. Overall, CNC had the highest thermal conductivity, followed by LB, and SDS and SDBS had similar thermal conductivity. Moreover, the heat capacity also had a similar value to thermal conductivity, as shown by acquiring the data on differences in temperature when the same quantity of heat was applied.

Therefore, in this study, it was found that cellulose is not only hydrophilic and eco-friendly, but it is also more thermally efficient than other surfactants. These data could be widely used for the base fluid for making nanofluids and in other industries.
